# Unknown mutations and genotype/phenotype correlations of autosomal recessive congenital ichthyosis in patients from Saudi Arabia and Pakistan

**DOI:** 10.1002/mgg3.539

**Published:** 2019-01-01

**Authors:** Dulce Lima Cunha, Omar Mohammed Alakloby, Robert Gruber, Naseebullah Kakar, Jamil Ahmad, Salem Alawbathani, Roswitha Plank, Katja Eckl, Birgit Krabichler, Janine Altmüller, Peter Nürnberg, Johannes Zschocke, Guntram Borck, Matthias Schmuth, Adnan S. Alabdulkareem, Kholood Abdulaziz Alnutaifi, Hans Christian Hennies

**Affiliations:** ^1^ Department of Biological and Geographical Sciences University of Huddersfield Huddersfield UK; ^2^ Division of Human Genetics Medical University of Innsbruck Innsbruck Austria; ^3^ Cologne Center for Genomics University of Cologne Cologne Germany; ^4^ Department of Dermatology, College of Medicine Imam Abdulrahman Bin Faisal University (formerly University of Dammam) Dammam Saudi Arabia; ^5^ Department of Dermatology Medical University of Innsbruck Innsbruck Austria; ^6^ Institute of Human Genetics University of Ulm Ulm Germany; ^7^ Department of Biotechnology BUITEMS Quetta Pakistan; ^8^ Department of Biology Edge Hill University Ormskirk UK; ^9^ CECAD Cluster of Excellence on Cellular Stress Responses in Aging‐associated Diseases University of Cologne Cologne Germany; ^10^ King Saud Medical City Riyadh Saudi Arabia

**Keywords:** congenital ichthyosis, erythema, genotype/phenotype correlation, homozygosity mapping, Skin permeability barrier, skin scaling

## Abstract

**Background:**

Autosomal recessive congenital ichthyosis (ARCI) is a genetically and phenotypically heterogeneous skin disease, associated with defects in the skin permeability barrier. Several but not all genes with underlying mutations have been identified, but a clear correlation between genetic causes and clinical picture has not been described to date.

**Methods:**

Our study included 19 families from Saudi Arabia, Yemen, and Pakistan. All patients were born to consanguineous parents and diagnosed with ARCI. Mutations were analyzed by homozygosity mapping and direct sequencing.

**Results:**

We have detected mutations in all families in five different genes: *TGM1*, *ABCA12*, *CYP4F22*, *NIPAL4*, and *ALOXE3*. Five likely pathogenic variants were unknown so far, a splice site and a missense variant in *TGM1*, a splice site variant in *NIPAL4*, and missense variants in *ABCA12* and *CYP4F22*. We attributed *TGM1 *and *ABCA12* mutations to the most severe forms of lamellar and erythematous ichthyoses, respectively, regardless of treatment. Other mutations highlighted the presence of a phenotypic spectrum in ARCI.

**Conclusion:**

Our results contribute to expanding the mutational spectrum of ARCI and revealed new insights into genotype/phenotype correlations. The findings are instrumental for a faster and more precise diagnosis, a better understanding of the pathophysiology, and the definition of targets for more specific therapies for ARCI.

## 1 INTRODUCTION

The skin barrier is imperative for protecting the organism from internal water loss as well as from outside pathogens and toxic compounds. Defects in the skin barrier function can lead to several skin disorders including autosomal recessive congenital ichthyosis (ARCI) (Feingold & Elias, 2014; Schmuth et al., 2013; Traupe, Fischer, & Oji, 2014).

Autosomal recessive congenital ichthyosis (MIM 242300, 242100, 606545, 601277, 242500, 604777, 612281, 615022, 613943, 615023, 602400, 615024, 617320, 617574, 617571) refers to a group of nonsyndromic congenital ichthyoses that include Harlequin ichthyosis (HI), lamellar ichthyosis (LI), and congenital ichthyosiform erythroderma (CIE) (Oji et al., 2010). Even though ARCI is a very heterogeneous disorder, common features include a generalized scaling of the skin, often with underlying erythema. Newborns usually present a collodion membrane that is lost during the first weeks of life. To date, 12 genes have been associated with ARCI: *TGM1* (MIM 190195), *ALOX12B* (MIM 603741), *ALOXE3* (MIM 607206), *ABCA12* (MIM 607800), *CYP4F22* (MIM 611495), *NIPAL4* (MIM 609383), *LIPN* (MIM 613924), *CERS3* (MIM 615276), *PNPLA1* (MIM 612121), *CASP14* (MIM 605848), *SDR9C7* (MIM 609769), and *SULT2B1* (MIM 604125), and at least 10%–15% of affected individuals do not have mutations in any of the known genes (Hellström Pigg et al., 2016; Vahlquist, Fischer, & Törmä, 2018). These genes have been linked to the maintenance of skin barrier function as their products are involved in the formation of the cornified lipid envelope in the stratum corneum and ceramide formation and processing in the epidermis (Eckl et al., 2013; Li, Loriè, Fischer, Vahlquist, & Törmä, 2012; Ohno et al., 2015).

HI is the most severe and often fatal form of the disease. Truncating mutations in *ABCA12* have been detected as the main cause for HI (Akiyama, 2014; Akiyama et al., 2005). LI and CIE, in contrast, have not yet been associated with a clear genotype/phenotype correlation. Patients with LI exhibit large, thick scales over the entire body without a severe background erythroderma. Patients with CIE normally present fine, whitish scales as well as generalized erythroderma. Both clinical forms can show partially overlapping phenotypes, ranging from coarse to fine scaling and mild to severe erythema (Rodriguez‐Pazos, Ginarte, Vega, & Toribio, 2013). In more than 50% of the patients, LI is caused by mutations in *TGM1,* however, *TGM1* mutations have also been reported to cause CIE (Becker et al., 2003; Herman et al., 2009), as well as other subtypes of ARCI, such as bathing suit ichthyosis (BSI) and self‐improving ichthyosis (SII) (Hackett, Fitzgerald, Watson, Hol, & Irvine, 2010; Raghunath et al., 2003). SII refers to a patient who is born with a collodion membrane but presents only a particularly mild phenotype after the first weeks of life.

Mutations in *ALOXE3 *and *ALOX12B*, that code for lipoxygenases eLOX‐3 and 12R‐LOX, and *NIPAL4* have been associated to LI, CIE and also SII (Eckl et al., 2009; Jobard et al., 2002; Vahlquist et al., 2010). Other ARCI genes include cytochrome P450 member *CYP4F22*, which encodes a fatty acid hydroxylase (Ohno et al., 2015), and defects of this gene have been linked to LI (Lefevre et al., 2006). Mutations in *CERS3, PNPLA1, SDR9C7,* and *SULT2B1*, have been identified in few families with ARCI presenting mainly LI phenotypes (Eckl et al., 2013; Grall et al., 2012; Heinz et al., 2017; Israeli et al., 2011; Shigehara et al., 2016).

We present the findings of a study that involved 19 consanguineous families from Saudi Arabia, Yemen, and Pakistan with affected members diagnosed with different forms of ARCI. For all of them, we have identified the underlying mutations causing the disease, using homozygosity mapping combined with Sanger sequencing. We have detected eleven different mutations in various ARCI‐associated genes, five of which are being reported for the first time.

## 2 MATERIALS AND METHODS

### 2.1 Ethical compliance

All procedures involving human participants were approved by institutional Research Ethics Committees and in accordance with the 1964 Helsinki declaration and its later amendments. Informed consent was obtained from all individual participants included in the study.

### 2.2 Patients and phenotypic features

**Figure 1:**
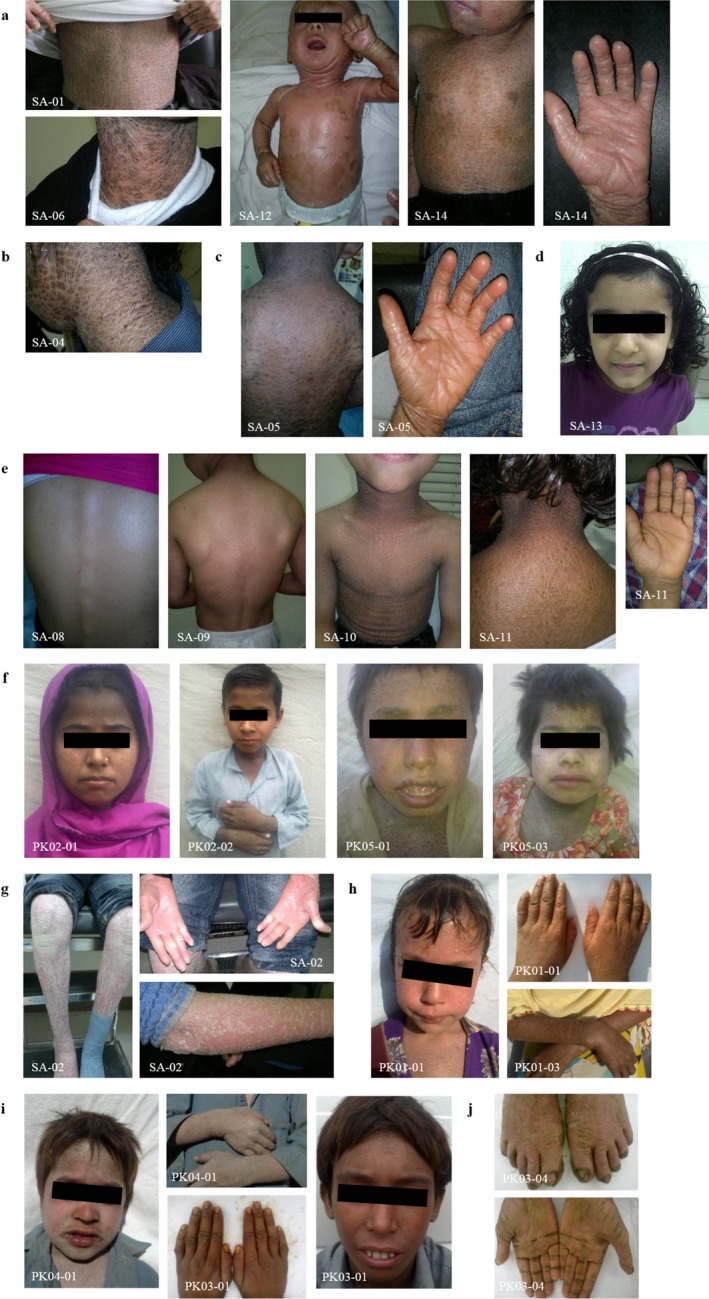
Clinical features of patients with ARCI, categorized by mutant genes. (a–c) *TGM1 *affected patients showed generalized severe, dark colored scales, and palmoplantar hyperkeratosis, apparently independent of type of mutation or treatment. (d) Patient diagnosed with SII, with no visible skin alterations at 6 years of age. (e–f) Patients with *NIPAL4* splice site (e) and missense (f) mutations presented a variable range of symptoms like milder to moderate scaling more prominent in the upper body half but no palmar hyperkeratosis. (g–h) Patients with *ABCA12 *mutations with fine to medium‐sized whitish scaling and erythema (SA‐02), and with slightly milder phenotype and white scales (PK01). (i) Patients from families PK04 and PK03 with generalized whitish scaling, quite visible on the hands, diagnosed with *ALOXE3* nonsense mutations. (j) Affected individuals from another branch of family PK03, initially also diagnosed with ARCI. Upon reinvestigation, they showed more localized severe scaling on the hands and feet as well as nail and teeth (not shown) anomalies consistent with a diagnosis of Papillon–Lefèvre syndrome caused by *CTSC* mutation

Samples from a total of 37 individuals diagnosed with different forms of ARCI and 72 unaffected members were collected. We have studied 19 consanguineous families, 13 from Saudi Arabia, 1 from Yemen, and 5 from Pakistan. At least one affected individual (index case) from each pedigree was tested by homozygosity mapping. The detailed clinical features of all index cases are shown in Table 1 and Figure 1.

**Table 1:** Patient information and clinical diagnosis

Patient	Gender	Birth year	Affected in family	Origin	Clinical diagnosis	Phenotypic features		Treatment
						Scaling	Erythema	
SA‐01	M	1997	1	Saudi Arabia	LI	Severe, generalized; coarse brown scales; palmoplantar hyperkeratosis	No	Acitretin (3–4 years, 11–20 mg)
SA‐02	M	1994	2	Saudi Arabia	CIE	Severe, generalized; fine white scales; palmoplantar hyperkeratosis	Severe	‐
SA‐04	F	1995	1	Saudi Arabia	LI	Severe generalized; coarse brown scales; palmoplantar hyperkeratosis	No	Acitretin (11–15 years, 21–30 mg)
SA‐05	F	2007	1	Saudi Arabia	LI	Severe generalized; coarse brown scales; palmoplantar hyperkeratosis	No	Acitretin (1–2 years, 5–10 mg)
SA‐06	F	1999	2	Saudi Arabia	LI	Severe generalized; coarse brown scales; palmoplantar hyperkeratosis	No	Acitretin (1–4 weeks, 5–10 mg)
SA‐08	F	1986	1	Saudi Arabia	LI	Mild generalized; fine light brown scales; plantar hyperkeratosis	Mild	Acitretin (5–6 years, 5–10 mg)
SA‐09	M	2003	2	Saudi Arabia	LI	Moderate generalized; fine light brown scales; plantar hyperkeratosis	No	NA
SA‐10	M	2003	2	Saudi Arabia	LI	Moderate generalized; coarse, large light brown scales; plantar hyperkeratosis	No	‐
SA‐11	F	2005	1	Saudi Arabia	LI	Severe generalized; coarse, large light brown scales; plantar hyperkeratosis	No	‐
SA‐12	M	2010	1	Saudi Arabia	LI	Severe generalized; coarse, large light brown, plate‐like scales; palmoplantar hyperkeratosis	No	‐
SA‐13	F	2006	2	Saudi Arabia	SII	No	No	‐
SA‐14	M	2006	1	Saudi Arabia	LI	Severe generalized scaling; coarse and large brown scales; palmoplantar hyperkeratosis	No	Acitretin (3–4 years, 5–10 mg)
SA‐15	M	2007	1	Saudi Arabia	LI	Moderate, more present in upper body; coarse, large light brown scales; plantar hyperkeratosis	No	‐
YE‐01	F	2006	1	Yemen	LI	Severe, generalized; coarse brown scales; palmoplantar hyperkeratosis	No	‐
PK01‐01	F	NA	4	Pakistan	CI	Generalized scaling, more visible in hands, neck and face; fine light‐colored scales	Moderate	NA
PK01‐02	M	NA						NA
PK02‐01	F	NA	2	Pakistan	CI	Scaling more severe in face, neck and hands; coarse brownish scales	No	NA
PK02‐02	M	NA						NA
PK03‐01	M	NA	2	Pakistan	CI	More visible in hands, neck and face; fine lighter‐colored, yellowish scales	Moderate	NA
PK03‐04	M	NA	4		PLS^a^	Severe localized in hands, feet, knees; nail and teeth malformation; transgredient hyperkeratosis	No	NA
PK04‐01	M	NA	1	Pakistan	CI	Scaling localized visible in hands, neck and face; lighter‐colored and less compact scales	Moderate	NA
PK05‐01	M	NA	5	Pakistan	CI	Scaling more severe in face, neck and hands; coarse brownish scales	No	NA
PK05‐03	F	NA						NA
PK05‐04	M	NA						NA

Note

CI: Congenital ichthyosis; CIE: Congenital ichthyosiform erythroderma; LI: Lamellar ichthyosis; NA: information not available; PLS: Papillon–Lefèvre syndrome; SII: Self‐improving ichthyosis.

a On reinvestigation following molecular analysis.

Affected individuals were clinically diagnosed with different forms of ARCI by experienced dermatologists and presented heterogeneous phenotypes. The majority of Saudi Arabian (SA) patients and the Yemenite (YE) patient showed a phenotype of LI, with generalized scaling and mild to no erythema. SA‐02 was the only patient with severe erythema and was hence diagnosed with CIE. SA‐13 was diagnosed with SII, as she presented no scaling or erythema but was reported to have had a collodion membrane at birth. Some patients were being treated with the retinoid acitretin, which modulates keratinocyte differentiation, at the time of the sample collections, but with different durations, ranging from few weeks to nearly 15 years of treatment.

Pakistani affected individuals (PK) showed more apparent generalized and severe localized scaling. The families had limited access to medical care and no pharmacological treatment options. Patients from families PK01 and PK04 presented moderate erythema. Family PK03 (Supporting information Figure S1) showed more dissimilar phenotypes among the patients: patient PK03‐01 had a more generalized but less severe scaling phenotype but patient PK03‐04 presented with more localized and severe scaling on hands and feet associated with visible tooth and nail malformations(Figure 1h,i).

### 2.3 Homozygosity mapping

Genomic DNA was extracted from peripheral blood or saliva samples with standard methods. Homozygosity mapping was performed in samples of index patients using HumanCytoSNP‐12 (Illumina, San Diego, CA, USA), following the manufacturer's instructions. Generated data were analyzed with Nexus Copy Number™ software (BioDiscovery, Hawthorne CA, USA). The longest regions of homozygosity were extracted for each index case and compared to the locations of known ARCI genes.

### 2.4 Sanger sequencing

Target exons were amplified by PCR using standard cycle conditions and in‐house designed primers (primers lists available upon request). Products were sequenced using BigDye Terminator v1.1 kit (Applied Biosystems, Foster City, CA, USA) and run on a Sequence Analyzer 3130XL (Applied Biosystems).

### 2.5 Next‐generation sequencing

Two index patients with a homozygous region matching *ABCA12* location were further analyzed with a custom‐made dermatogenetics gene panel comprising 56 genes including all known ARCI genes using an Illumina HiSeq sequencing system (Illumina).

Because no homozygous mutation was found in any known ARCI gene in PK03‐04, in contrast to his nephew PK03‐01, exome sequencing was performed using enrichment with SureSelect Human All Exon V6 (Agilent, Santa Clara, CA) and a HiSeq sequencing system (Illumina). Data analysis of filter‐passed reads was done with BWA‐short in combination with GATK and SAMTOOLS as implemented in the in‐house analysis tool Varbank (Cologne Center for Genomics).

### 2.6 In silico data analysis

Sequences were analyzed with SeqmanPro (DNASTAR Inc., Madison, WI, USA). Unknown missense variants were regarded as likely pathogenic if they were predicted to be damaging by at least two of the algorithms MutTaster (Schwarz, Cooper, Schuelke, & Seelow, 2014), SIFT (Kumar, Henikoff, & Ng, 2009) and PolyPhen‐2 (Adzhubei et al., 2010), affected highly conserved amino acids, and were not found as homozygous variants in control DNA sequences as analyzed by the Exome Aggregation Consortium (exac.broadinstitute.org) and the 1,000 Genomes Project (www.internationalgenome.org). Splice site variants were analyzed with Human Splicing Finder 3.0 (Desmet et al., 2009). Protein domains were determined using PFAM (Finn, Coggill, Eberhardt, & Eddy, 2016).

### 2.7 GenBank Accession numbers

***TGM1*: **NM_00359.2/NP_000350.1

***NIPAL4*: **NM_001099287.1/NP_001092757.1

***ABCA12***: NM_173076.2/NP_775099.2.

***CYP4F22***: NM_173483.3/NP_775754.2.

***ALOXE3***: NM_001165960.1/NP_001159432.1

***CTSC***: NM_001814.4/NP_001805.3

## 3 RESULTS

DNA samples from 13 unrelated ARCI families from Saudi Arabia, one from Yemen as well as five extended families from Pakistan were used in this study to investigate the genetic causes of ARCI. After aligning the location of known ARCI genes with regions of homozygosity obtained from genome‐wide homozygosity mapping, one to three candidate intervals containing one or two of these genes each were found for all index cases (Supporting information Table S1). Mutations were identified with conventional direct sequencing of candidate genes, except for *ABCA12*, which comprises 53 exons and was therefore analyzed with gene panel sequencing. Homozygous causal mutations were found for each index case, and co‐segregation of mutations with phenotypes was confirmed in all available family members. Previously unknown variants in ARCI genes were assessed in silico and classified as defined in Methods. A summary of the mutations is shown in Table 2.

**Table 2:** Mutations identified in Saudi Arabian and Pakistani families

Patient	Gene^a^	Mutation cDNA^b^	Mutation protein	Prediction (SpliceFinder, Polyphen, SIFT, MutTaster)	MAF^c^	References
SA‐01	*TGM1*	c.398_407dupAGTATGAGTA	p.(Tyr136*)	Premature termination	0.000004^d^	Wakil et al. (2016)
SA‐02	*ABCA12*	**c.4541G>T**	**p.(Arg1514Leu)**	Damaging	‒	This study
SA‐04	*TGM1*	**c.1340A>C**	**p.(Asp447Ala)**	Damaging	‒	This study
SA‐05	*TGM1*	**c.758‐1G>C**	‐	Aberrant splicing 100%	‒	This study
SA‐06	*TGM1*	c.398_407dupAGTATGAGTA	p.(Tyr136*)	Premature termination	0.000004^d^	Wakil et al. (2016)
SA‐08	*NIPAL4*	**c.223+5_223+22delGTACGGCAGGGCTGGGGA**	‐	Aberrant splicing 61.4%	‒	This study
SA‐09	*NIPAL4*	**c.223+5_223+22delGTACGGCAGGGCTGGGGA**	‐	Aberrant splicing 61.4%	‒	This study
SA‐10	*NIPAL4*	**c.223+5_223+22delGTACGGCAGGGCTGGGGA**	‐	Aberrant splicing 61.4%	‒	This study
SA‐11	*NIPAL4*	**c.223+5_223+22delGTACGGCAGGGCTGGGGA**	‐	Aberrant splicing 61.4%	‒	This study
SA‐12	*TGM1*	c.398_407dupAGTATGAGTA	p.(Tyr136*)	Premature termination	0.000004^d^	Wakil et al. (2016)
SA‐13	*TGM1*	c.871G>A	p.(Gly291Ser)	Damaging	0.00001	Sakai et al. (2016)
SA‐14	*TGM1*	c.398_407dupAGTATGAGTA	p.(Tyr136*)	Premature termination	0.000004^d^	Wakil et al. (2016)
SA‐15	*CYP4F22*	**c.982G>A**	**p.(Glu328Lys)**	Damaging	0.000004	This study
YE‐01	*TGM1*	c.398_407dupAGTATGAGTA	p.(Tyr136*)	Premature termination	0.000004^d^	Wakil et al. (2016)
PK01‐01 PK01‐02 PK01‐04	*ABCA12*	c.4676G>T	p.(Gly1559Val)	Damaging	0.000004	Nawaz et al. (2012)
PK02‐01 PK02‐02	*NIPAL4*	c.527C>A	p.(Ala176Asp)	Damaging	0.0007	Lefevre et al. (2004)
PK03‐01 PK03‐04	*ALOXE3* *CTSC*	c.814C>T c.901G>A	p.(Arg272*) p.(Gly301Ser)	Premature termination	0.00001 0.00003	Ullah et al. (2016) Toomes et al. (1999)
PK04‐01	*ALOXE3*	c.814C>T	p.(Arg272*)	Premature termination	0.00001	Ullah et al. (2016)
PK05‐01 PK05‐03 PK05‐04	*NIPAL4*	c.527C>A	p.(Ala176Asp)	Damaging	0.0007	Lefevre et al. (2004)

a *TGM1*: NM_00359.2; *ABCA12*: NM_173076.2; *NIPAL4*: NM_001099287.1; *CYP4F22*: NM_173483.3 *ALOXE3*: NM_001165960.1; *CTSC*: NM_001814.4.

b Mutations first identified in this study are shown in bold.

c Minor allele frequency (MAF) according to the Genome Aggregation Database (gnomad.broadinstitute.org).

d Same codon change resulting from a different variant.

### 3.1 Families from Saudi Arabia and Yemen

**Figure 2:**
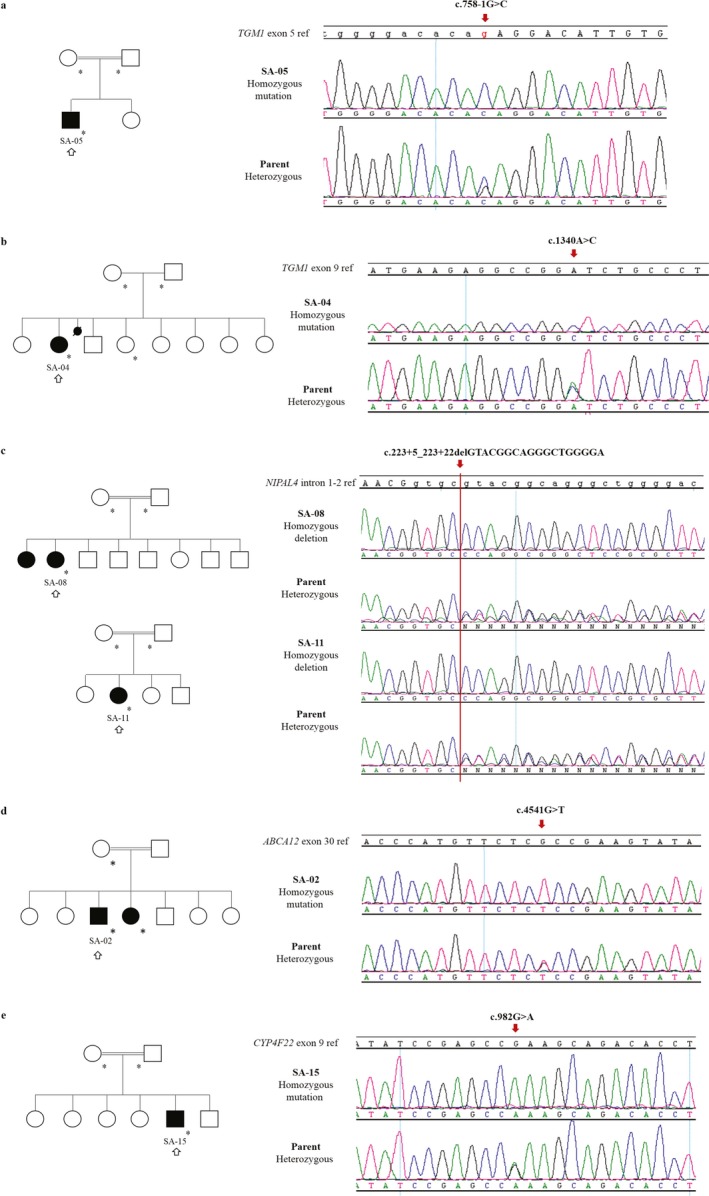
Family pedigrees and sequences of unknown mutations identified in this study. Pedigrees show index patients used for homozygosity mapping marked with a black outlined arrow (↑) and all samples confirmed by Sanger sequencing marked with a star (*). All parents of patients have a consanguinity relation. At least one parent is included in the displayed sequences to demonstrate co‐segregation. Red arrows indicate the positions of nucleotide substitutions or deletions. In the reference sequences, uppercase letters indicate exon nucleotides and lowercase letters intronic bases. (a) Examples of two Saudi Arabian pedigrees with patients with a homozygous intronic deletion in intron 1 of *NIPAL4*. (b) A homozygous missense mutation c.1340A>C was detected in exon 9 of *TGM1 *in SA‐04. (c) Patient SA‐05 was identified with a homozygous mutation in the acceptor splice site of *TGM1 *intron 4. (d) A previously unknown missense mutation in exon 9 of *CYP4F22* was found in SA‐15

Mutations in *TGM1 *were the most common in this study, found in seven of the thirteen index patients from Saudi Arabia. Aside from SA‐13 diagnosed with SII, all patients with *TGM1* mutations had been diagnosed with LI and presented with similar clinical features (Figure 1a‐d). SA‐01, SA‐06, SA‐12, SA‐14, and YE‐01 had the same homozygous duplication c.398_407dupAGTATGAGTA in exon 3, which leads to a premature stop codon (p.Tyr136*). This mutation has been linked to two ARCI families from Saudi Arabia (Wakil et al., 2016), pointing to a potential founder mutation on the Arabian Peninsula. SA‐04, also diagnosed with LI, presented a missense change in exon 9, c.1340A>C (p.Asp447Ala). This variant is located within the catalytic core of transglutaminase 1 (Figure 2b). A splice acceptor site change c.758‐1G>C in intron 4 of *TGM1* was found in SA‐05 (Figure 2a). Patient SA‐13, diagnosed with SII based on the lack of signs for congenital ichthyosis aside from the presence of a collodion membrane at birth, presented a homozygous missense variant in exon 5 of *TGM1*, c.871G>A (p.Gly291Ser). This variant was previously reported in a Japanese LI patient with compound heterozygosity, which might explain the different forms of the disease (Sakai et al., 2009). This finding is in accordance with Hackett et al, who reviewed the phenotypes associated with more than 40 mutations reported in *TGM1* and noted that mutations associated with SII seemed to cluster in exons 5, 6, and 7 of *TGM1* (Hackett et al., 2010).

Four patients diagnosed with LI (SA‐08, SA‐09, SA‐10, and SA‐11) presented a common, previously unreported homozygous intronic deletion of 18 nucleotides in *NIPAL4*, c.223+5_223+22delGTACGGCAGGGCTGGGGA, which is predicted to affect the donor splice site of intron 1 (Figure 2c).

SA‐02, a patient diagnosed with CIE, was found to have a homozygous missense variant, c.4541G>T (p.Arg1514Leu), in exon 30 of *ABCA12* (Figure 2d). SA‐15, diagnosed with LI, has a missense variant c.982G>A (p.Glu328Lys) in exon 9 of *CYP4F22*, which has not been described before (Figure 2e). This variant was predicted as deleterious and is located inside the predicted cytochrome P450 domain.

### 3.2 Families from Pakistan

We found a homozygous missense variant in exon 31 of *ABCA12*, c.4676G>T (p.Gly1559Val), in family PK01. Nawaz *et al* described this variant before in a consanguineous family from Pakistan with non‐bullous CIE (Nawaz et al., 2012).

Patients from families PK02 and PK05 were identified with the same homozygous missense variant c.527C>A (p.Ala176Asp) in exon 4 of *NIPAL4*. This variant was reported before (Lefevre et al., 2004) and is located within the suggested transporter domain of the NIPAL4 protein.

A homozygous nonsense mutation, c.814C>T (p.Arg272*), in exon 4 of *ALOXE3* was found in patients of family PK04. The mutation was reported in a Pakistani family diagnosed with CIE (Ullah et al., 2016).

Homozygosity mapping in index cases of family PK03 (Supporting information Figure S1) showed a homozygous region matching the *ALOXE3/ALOX12B* genes location, and Sanger sequencing revealed the same homozygous mutation as seen in PK04, c.814C>T (p.Arg272*) in *ALOXE3*, in one branch of the family. However, affected individuals from another branch were either heterozygous for the mutation or even homozygous for the reference allele. Reinvestigation demonstrated that these patients were also affected with nail malformation and early loss of teeth and the skin lesions were more limited to the extremities, in contrast to PK03‐01 and his affected sister. PK03‐04 was then analyzed by exome sequencing and the likely pathogenic variant c.901G>A (p.Gly301Ser) in *CSTC,* the gene encoding cathepsin C, was found.

## 4 DISCUSSION

Using a combination of homozygosity mapping and candidate gene sequencing, we identified eleven different mutations in five genes in a total of 19 consanguineous families with ARCI from Saudi Arabia, Yemen, and Pakistan. All variants were classified as pathogenic or likely pathogenic and were homozygous as expected, and thus represent a particularly useful resource to search for genotype/phenotype correlations, although the clinical appearance was different between Saudi Arabian and Pakistani patients based on the level of medical care available. All patients with *TGM1* mutations, except SII‐diagnosed SA‐13, presented the most severe phenotypes, with generalized coarse brown scaling (Figure 1a–d). Interestingly, this phenotype seems independent of the type of mutation and even prominent after oral treatment with acitretin. Two unknown disease‐causing mutations were found in *TGM1*: p.Asp447Ala (c.1340A>C) in exon 9 and c.758‐1G>C at the splice acceptor site of intron 4. Even after treatment with acitretin for approximately 15 years, the patient with mutation p.Asp447Ala presented a severe phenotype, similar to non‐treated patients with *TGM1* mutations. The second mutation was found in a patient with LI and a similar phenotype with severe scaling and rough and brownish scales.

In contrast, the patients from the Saudi Arabian cohort carrying the same *NIPAL4* intronic deletion, c.223+5_223+22delGTACGGCAGGGCTGGGGA, showed variable phenotypes, ranging from mild to severe generalized scaling, with white to light brown‐colored scales, fine or coarse. SA‐08 had the mildest symptoms, with mild scaling and skin dryness, likely improved by the treatment with acitretin. On the other hand, SA‐11 was never treated with acitretin and presented the most severe phenotype in this group, with coarse light brown scales and severe scaling and dryness (Figure 1e). Patient SA‐15 with a *CYP4F22* mutation, also diagnosed with LI, presented moderate skin dryness and more localized scaling in the face, neck, abdomen, back, and arms (photos not available).

SA‐02, diagnosed with CIE and with the unreported *ABCA12 *missense mutation p.Arg1514Leu (c.4541G>T), presented a severe skin phenotype, including generalized severe scaling with fine white scales, pronounced erythema and palmoplantar keratoderma (Figure 1g). A homozygous G to A change at the same position leading to p.Arg1514His was described in ARCI before (Lefevre et al., 2003).

Notably, we identified two autosomal recessive skin disorders in the same consanguineous family, PK03, initially diagnosed only with ARCI. A homozygous nonsense mutation in exon 4 of *ALOXE3* was found in index patient PK03‐01 and segregation was confirmed by analysis of the parents and siblings. The phenotype of PK03‐01 also matched the phenotype of PK04‐01 who showed the same mutation (Figure 1h). However, the index patient PK03‐04 of the second branch of the family and his affected siblings, initially diagnosed with ARCI, showed localized scaling mostly on hands and feet associated with nail malformations and teeth loss at an early age on reinvestigation (Figure 1i). They did not carry the homozygous *ALOXE3* mutation, and exome sequencing revealed a homozygous mutation in *CTSC*, which was previously reported in patients with Papillon–Lefèvre syndrome (PLS) (Nagy et al., 2014; Noack et al., 2008; Toomes et al., 1999). PLS (MIM #245000) is a rare autosomal recessive condition presenting diffuse keratoderma together with rapidly progressive periodontitis (Dhanrajani, 2009). This finding confirmed two different diagnoses of ARCI and PLS, respectively, in two branches of the family (Supporting information Figure S1).

**Figure 3:**
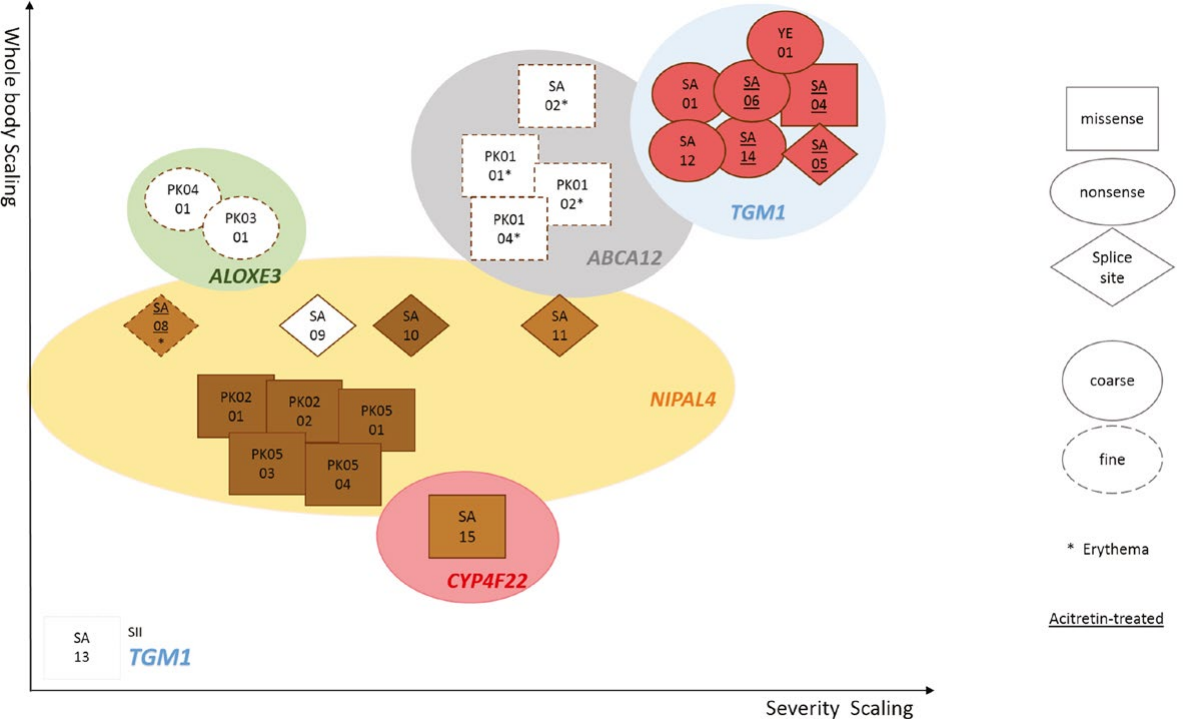
Schematic representation of genotype/phenotype correlations of ARCI found in this study. All phenotypes and genotypes were grouped according to our findings. Each index case is named and boxed according to the severity of the scaling (X axis) and its extension (Y axis). Types of mutations are indicated by the shape of the boxes, that is, squares for missense, oval for nonsense, and diamonds for splice site mutations. Scaling type was also included, with coarse scales represented by a full‐outline box and fine scales by dashed outlines. Patients with an erythematous phenotype are represented by an asterisk (*) and those treated with acitretin are underlined

The identification of various homozygous mutations in different genes facilitated an important contribution to defining a genotype/phenotype correlation for ARCI (Figure 3). Mutations in *TGM1 *and *ABCA12* cause the most severe phenotypes, compared to *NIPAL4, CYP4F22, *and *ALOXE3 *mutations. We could also establish parameters regarding the color and appearance of the scales, where patients with defects in *TGM1* seem to present the darkest and coarsest scales, followed by *CYP4F22*, *NIPAL4* and lastly *ALOXE3*, which ranged from light brown to white. *ABCA12 *affected patients presented the lightest colored scales, all of them white and fine. Furthermore, all Saudi Arabian patients showed palmar hyperlinearity but only *TGM1* and *ABCA12* affected individuals presented palmoplantar hyperkeratosis, while *NIPAL4* and *CYP4F22* presented only plantar keratosis.

Our study demonstrated that homozygosity mapping coupled with Sanger sequencing is still a valid and cost‐efficient tool to identify rare disease‐causing mutations in consanguineous families. While establishing a genotype/phenotype correlation for ARCI patients is a difficult task, our findings have added strong and new insights to this matter, hopefully leading to a better clinical and molecular understanding of this heterogeneous disease and to novel approaches for pathophysiology‐based therapies.

## CONFLICT OF INTEREST

The authors have no potential conflict of interest to declare.
