# Supplementary material for: Unknown mutations and genotype/phenotype correlations of autosomal recessive congenital ichthyosis in patients from Saudi Arabia and Pakistan
Source: Mol Genet Genomic Med. 2019 Jan 1;7(3):e539. doi: 10.1002/mgg3.539 (PMC6418373; doi:10.1002/mgg3.539)
Supplement: Supplementary file 1 [file MGG3-7-na-s001.pdf]

Unknown mutations and genotype/phenotype correlations of autosomal recessive congenital ichthyosis in patients from Saudi Arabia and Pakistan

Journal Molecular Genetics & Genomic Medicine

Authors Dulce Lima Cunha[1,2,3] [ORCID 0000-0002-6814-8365], Omar Mohammed Alakloby[4], Robert Gruber[5], Naseebullah Kakar[6,7], Jamil Ahmad[7], Salem Alawbathani[3], Roswitha Plank[1,2], Katja Eckl[1,2,8], Birgit Krabichler[2], Janine Altmüller[3], Peter Nürnberg[3,9], Johannes Zschocke[2], Guntram Borck[6], Matthias Schmuth[5], Adnan S. Alabdulkareem[10], Kholood Abdulaziz Alnutaifi[4], Hans Christian Hennies[1,2,3,9] [ORCID 0000-0001-7210-2389]

Corresponding Author Dr. Hans C. Hennies, Dept. of Biological and Geographical Sciences, University of Huddersfield, Huddersfield HD1 3DH, UK; phone +44-1484-473014; email h.c.hennies@hud.ac.uk

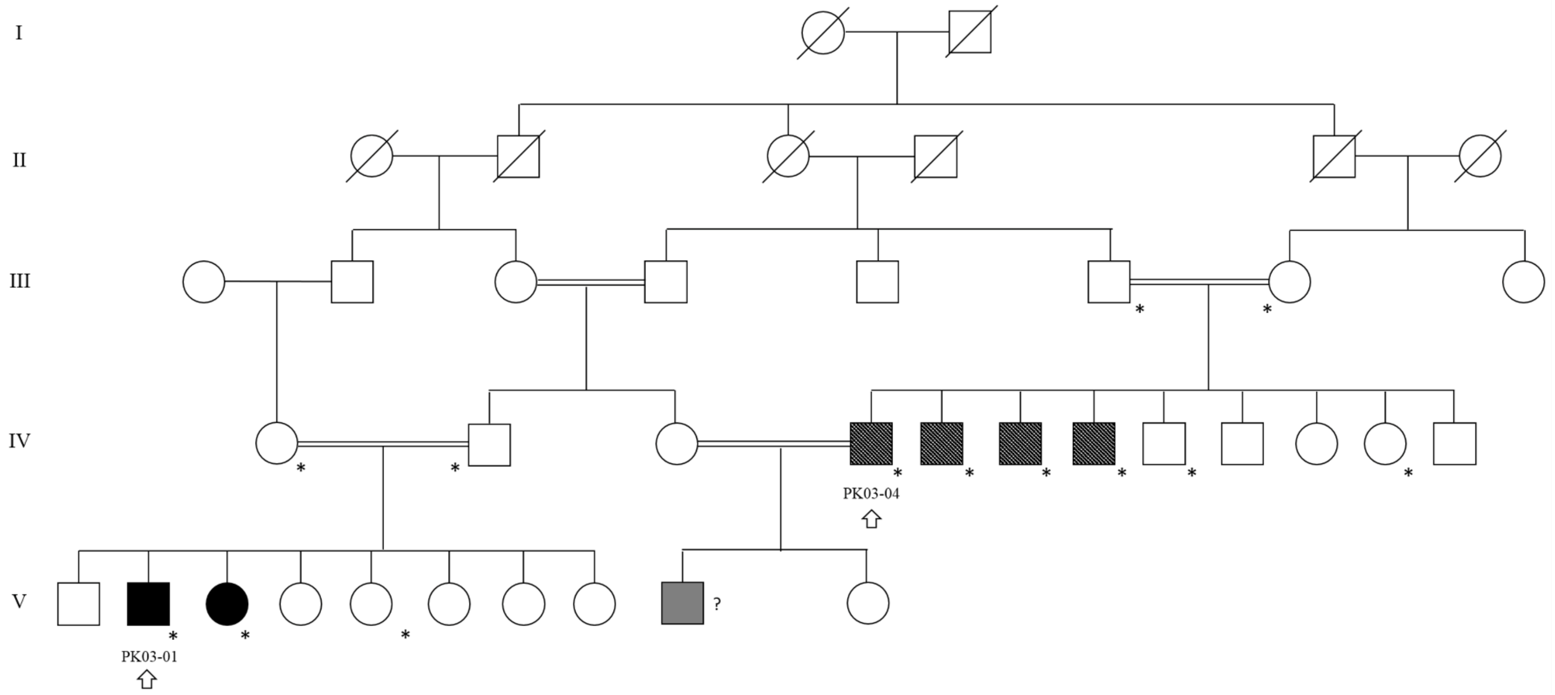

Figure S1. Pedigree of family PK03. Mutation analysis confirmed a diagnosis of ARCI caused by mutations in *ALOXE3* in the left branch (filled symbols) and revealed Papillon Lefèvre syndrome caused by mutations in *CTSC* in the right branch (hatched symbols) of the family. One reportedly affected person was not seen by any of the authors (grey symbol). Index cases marked by arrows (⇧) were analysed with homozygosity mapping, co-segregation was confirmed in individuals marked by asterisks (*).
