# Supplementary material for: Unknown mutations and genotype/phenotype correlations of autosomal recessive congenital ichthyosis in patients from Saudi Arabia and Pakistan
Source: Mol Genet Genomic Med. 2019 Jan 1;7(3):e539. doi: 10.1002/mgg3.539 (PMC6418373; doi:10.1002/mgg3.539)
Supplement: Supplementary file 2 [file MGG3-7-na-s002.pdf]

Unknown mutations and genotype/phenotype correlations of autosomal recessive congenital ichthyosis in patients from Saudi Arabia and Pakistan

Journal Molecular Genetics & Genomic Medicine

Authors Dulce Lima Cunha[1,2,3] [ORCID 0000-0002-6814-8365], Omar Mohammed Alakloby[4], Robert Gruber[5], Naseebullah Kakar[6,7], Jamil Ahmad[7], Salem Alawbathani[3], Roswitha Plank[1,2], Katja Eckl[1,2,8], Birgit Krabichler[2], Janine Altmüller[3], Peter Nürnberg[3,9], Johannes Zschocke[2], Guntram Borck[6], Matthias Schmuth[5], Adnan S. Alabdulkareem[10], Kholood Abdulaziz Alnutaifi[4], Hans Christian Hennies[1,2,3,9] [ORCID 0000-0001-7210-2389]

Corresponding Author Dr. Hans C. Hennies, Dept. of Biological and Geographical Sciences, University of Huddersfield, Huddersfield HD1 3DH, UK; phone +44-1484-473014; email h.c.hennies@hud.ac.uk

Table S1. Homozygosity regions matching ARCI gene intervals identified after homozygosity mapping.

Patient	Chromosome region	% Heterozygosity	Length	Cytoband	ARCI gene match
SA-01	chr14:23,172,456-25,025,737	0.0	1,853,282	14q11.2 - q12	*TGM1*
SA-02	chr2:202,252,090-215,853,470	0.304	13,601,381	2q33.1 - q35	*ABCA12*
SA-04	chr14:22,879,230-28,961,059	0.630	6,081,830	14q11.2 - q12	*TGM1*
SA-05	chr14:23,172,456-25,203,263	0.0	2,030,808	14q11.2 - q12	*TGM1*
SA-06	chr2:175,291,934-220,342,826	0.172	45,050,893	2q31.1 - q35	*ABCA12*
	chr14:19,283,777-29,454,152	0.283	10,170,376	14q11.2 - q12	*TGM1*
SA-08	chr5:148,741,029-168,469,088	0.0	19,728,060	5q33.1 - q35.1	*NIPAL4*
SA-09	chr5:140,154,033-161,846,378	0.118	21,692,346	5q31.3 - q34	*NIPAL4*
	chr15:91,645,431-100,338,915	0.0	8,693,485	15q26.1 - q26.3	*CERS3*
SA-10	chr5:141,611,117-169,435,977	0.0	27,824,861	5q31.3 - q35.1	*NIPAL4*
SA-11	chr5:123,557,892-159,370,345	0.072	35,812,454	5q23.2 - q33.3	*NIPAL4*
SA-12	chr14:21,340,759-32,111,277	0.105	10,770,519	14q11.2 - q13.1	*TGM1*
SA-13	chr14:19,283,777-32,590,349	0.226	13,306,573	14q11.2 - q13.1	*TGM1*
SA-14	chr6:25,601,239-50,108,951	0.037	24,507,713	6p22.2 - p12.3	*PNPLA1*
	chr17:6,908,440-14,157,308	0.089	7,248,869	17p13.1 - p12	*ALOXE3/ALOX12B*
	chr14:20,866,317-25,502,331	0.0	4,636,015	14q11.2 - q12	*TGM1*
SA-15	chr19:15,434,569-22,399,457	0.206	6,964,889	19p13.12 - p12	*CYP4F22*
YE-01	chr14:24,679,877-32,924,012	0.77	8,244,135	14q11.2 - q12	*TGM1*
PK01-01<br>PK01-02<br>PK01-04	chr2:179,263,031-220,406,107	aggregate of 3 patients<br>NA	41,143,076	2q31.2 - q35	*ABCA12*
PK02-01<br>PK02-02	chr2:186,002,691-223,060,424<br>chr5:149,594,981-163,460,290	aggregate of 2 patients<br>NA	37,057,733<br>13,865,309	2q32.1 - q36.1<br>5q33.1 - q34	*ABCA12*<br>*NIPAL4*
PK03-01<br>PK03-04	chr17:770,461-13,845,956	aggregate of 2 patients<br>NA	13,075,495	17p13.3 - p12	*ALOXE3/ALOX12B*
PK04- 01	chr17:5,757,535-13,141,886	0.094	7,384,352	17p13.2 - p12	*ALOXE3/ALOX12B*
PK05-01<br>PK05-03<br>PK05-04	chr5:149,650,749-166,821,863	aggregate of 3 patients<br>NA	17,171,114	5q33.1 - q34	*NIPAL4*
